# Developing a novel causal inference algorithm for personalized biomedical causal graph learning using meta machine learning

**DOI:** 10.1186/s12911-024-02510-6

**Published:** 2024-05-27

**Authors:** Hang Wu, Wenqi Shi, May D. Wang

**Affiliations:** 1 Coulter Department of Biomedical Engineering, Georgia Insitute of Technology, Atlanta, USA; 2 Department of Electrical and Computer Engineering, Georgia Insitute of Technology, Atlanta, USA

**Keywords:** Causal inference, Precision medicine, Meta-learning, Causal graph learning

## Abstract

**Background:**

Modeling causality through graphs, referred to as causal graph learning, offers an appropriate description of the dynamics of causality. The majority of current machine learning models in clinical decision support systems only predict associations between variables, whereas causal graph learning models causality dynamics through graphs. However, building personalized causal graphs for each individual is challenging due to the limited amount of data available for each patient.

**Method:**

In this study, we present a new algorithmic framework using meta-learning for learning personalized causal graphs in biomedicine. Our framework extracts common patterns from multiple patient graphs and applies this information to develop individualized graphs. In multi-task causal graph learning, the proposed optimized initial guess of shared commonality enables the rapid adoption of knowledge to new tasks for efficient causal graph learning.

**Results:**

Experiments on one real-world biomedical causal graph learning benchmark data and four synthetic benchmarks show that our algorithm outperformed the baseline methods. Our algorithm can better understand the underlying patterns in the data, leading to more accurate predictions of the causal graph. Specifically, we reduce the structural hamming distance by 50-75%, indicating an improvement in graph prediction accuracy. Additionally, the false discovery rate is decreased by 20-30%, demonstrating that our algorithm made fewer incorrect predictions compared to the baseline algorithms.

**Conclusion:**

To the best of our knowledge, this is the first study to demonstrate the effectiveness of meta-learning in personalized causal graph learning and cause inference modeling for biomedicine. In addition, the proposed algorithm can also be generalized to transnational research areas where integrated analysis is necessary for various distributions of datasets, including different clinical institutions.

## Introduction

Causal relationship discovery between variables is one of the fundamental problems in biomedical research, and clinical practice [1]. For instance, inferring gene regulatory networks (GRNs) [2] from gene expression data can help uncover causal relationships such as inhibition and promotion among genes and protein targets, while observing fMRI imaging can reveal causal links between components of neuron networks [3]. Epidemiological research also benefits from causal discovery in studying the link between a disease outcome and its risk factors [4]. Understanding causal relationships has numerous practical benefits, including improved experimentation design, biomarker identification, and drug discovery [2]. Causal inference is focused on establishing cause-and-effect connections to reflect the inherent and universal interdependence of variables and reveal consistent causal relationships across various contexts [5].

In clinical decision-making, physicians typically reason from a cause-and-effect perspective, focusing on the causes of diseases and treatment outcomes. However, many machine learning models used in clinical decision support systems rely on predicting correlations among variables of interest [1]. For example, in patients with high cholesterol, we might calculate the percentage of patients with high cholesterol who do not exercise regularly or estimate the likelihood of a patient having high cholesterol based on observational data such as age and exercise level. These calculations are based on correlations; they do not reflect the causes of high cholesterol in each patient. In addition, understanding the causal effect is essential for personalized and precise treatment recommendations. For instance, high cholesterol in one patient might be caused by obesity, while in another patient, it could be caused by age, genetic predisposition, or lack of exercise. To provide individualized treatment plans, clinicians need to identify the causes of high cholesterol in each patient. To this end, causal machine learning can use observational data and intervention information to (1) quantify the causal effect for individuals and (2) infer the underlying causal structure for populations in biomedicine.

**Fig. 1:**
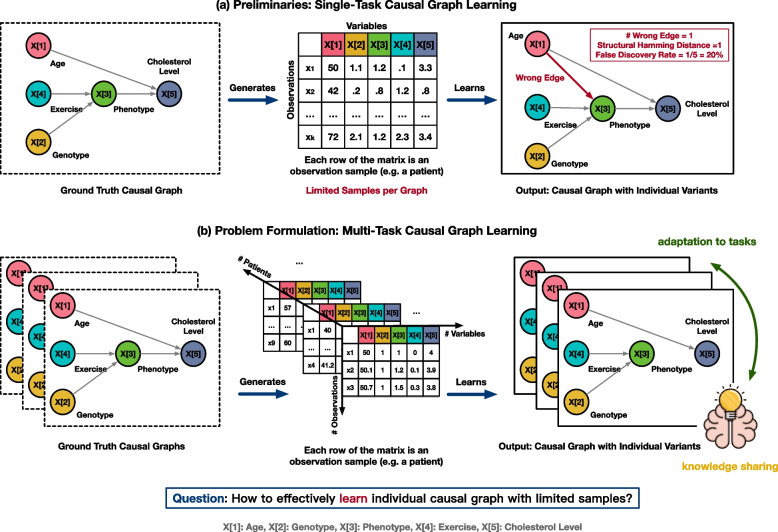
Problem formulation of multi-task causal graph learning in biomedicine. There are two scenarios of causal graph learning: **a** single-task (patient) setting and **b** multi-task setting. Each patient has a unique causal graph and his or her own observational data matrix. There are three dimensions to the data: the number of variables in the causal graphs, the number of patients/tasks, and the number of observational data points for each patient. Ideally, to deliver personalized medicine, it is important to construct a personalized causal graph for each patient. This study treats learning the causal graph for each patient as a single task and concentrates on multi-task settings. Although conventional approaches treat each task independently, we propose a novel causal inference approach to extract knowledge shared across all given tasks and to use meta-learning to enable rapid adaptation to new tasks

Causal inference graphs are commonly used to establish these causal relationships in order to make correct inferences about variable relationships. For example, Fig. 1a shows an example causal graph among the five variables: age, genotype A, phenotype B, exercise level, and cholesterol level. Each node in the graph corresponds to one of these variables, and each directed edge from node *X*[*i*] to node *X*[*j*] indicates the existence of a causal relationship from the variable *X*[*i*] to the variable *X*[*j*]. In addition to the qualitative causal relationships described by the existence of edges, we also focus on quantitative relationships between a variable and its parent variables. In the same example in Fig. 1a, for the cholesterol level, one possible functional relationship is *cholesterol level = - exercise level + age / 50 + genotype A + phenotype B*. The gold standard for constructing such causal graphs is to perform intervention experiments, i.e., changing the value of a single variable to observe changes in other variables. However, intervention experiments can be expensive and difficult to control, making it challenging to define interventions for all possible variable combinations. An alternative approach is to use existing observational data to learn causal relationships among available variables. For each patient, we record the values of variables at different time points, resulting in an observational data matrix, where each row represents a sample observation, and each column corresponds to one variable. Given this data matrix as a starting point, we can learn a causal graph with the highest likelihood of having generated this matrix. This method, known as causal graph learning with observational data, determines the causal relationships between all possible pairs of variable combinations. With observational data, we can search for a graph that maximizes the observed data likelihood subject to certain graph constraints.

In biomedicine, the genotype and phenotype of each patient result from a unique causal graph. For example, in Fig. 1a and b, we show two different causal graphs of the five variables for two individual patients. For heterogeneous biomedical data, they share some *commonalities*: they both contain edges from genotype A to phenotype B. Nevertheless, each of the graphs exhibits its own set of distinctive characteristics: phenotype B is shown to have an effect on the cholesterol level of this patient in (a), whereas there is no causal association between phenotype B and the cholesterol level in (b). As a result, variations in causal structures make it challenging to extract commonalities from existing data for the adoption of new tasks. In addition, learning all relationships for each causal graph requires a large amount of observational data [6]. However, in biomedicine, there are often limited observations for each patient, which makes learning personalized causal graphs for a collection of patients a very challenging computational problem. To summarize, biomedical causal graph learning problems present two distinct obstacles: (1) variation between causal graphs and (2) sample size limitations per graph.

In this paper, we develop a novel algorithmic framework for learning personalized causal graphs for multiple patients using meta-machine learning. Intuitively, although we have only limited samples per patient (task), integrating them together may be sufficient to learn the shared network interactions (i.e., commonalities). Once we have learned the shared network structures across all patients, we can learn individual variations for each patient. The combination of shared and individual topology can aid in the construction of personalized causal graphs. To capture our intuition algorithmically, we define each task of causal graph learning as a maximum likelihood problem of the graph structure. We assume that each task (i.e., learning a causal graph for a given patient) is drawn from a fixed underlying distribution. Learning the commonality shared among all tasks is learning characteristics of the distribution while learning variations of each task is learning an individual point from the distribution. In solving each task, we start from an initial guess of the graph structure, which corresponds to the initial value of an optimization problem. Solving the maximum likelihood problem can be regarded as adding/deleting edges and tuning the functional relationships of each edge so that the likelihood of observing the data is maximized. We use the initial guess to characterize the shared commonality of all graphs: if the initial guess is accurate enough, we may be able to acquire a better understanding of the personalized graph using the limited samples available for the task. The objective of our learning framework is to identify a good initial guess of the shared commonality by iteratively updating this guess. The contribution of our method is three-fold:

- We propose a novel causal inference framework to learn personalized biomedical causal graphs with a limited sample size per graph. By employing a meta-learning framework, we enable accurate personalized causal graph learning by sharing knowledge sharing across a collection of correlated causal structure learning tasks.
- In multi-task causal graph learning, the optimized initial guess of shared commonality enables the rapid adoption of knowledge to new tasks for efficient causal graph learning.
- Extensive experiments demonstrate the accuracy and efficiency of the proposed causal structure learning framework, comparing with baselines. To the best of our knowledge, this is the first study to demonstrate the effectiveness of meta-learning in personalized causal graph learning and cause inference modeling for biomedicine.

## Related works

### Causal discovery in biomedicine with heterogeneous data

Causal learning problems can be solved via maximum likelihood estimation techniques, for example, an L1-regularized maximum likelihood. Another approach is the recent state-of-the-art graph learning algorithm proposed by Zhang et al. [7], which constrains the graph from being directed acyclic. As biomedical datasets are heterogeneous and collected under various conditions, researchers have started to explore the importance of integrating multiple datasets for causal discovery. For example, Rau et al. [8] used a Markov Chain Monte Carlo sampling algorithm and showed that incorporating additional perturbation datasets helps identify the true underlying causal graph. They found that in the case of gene regulatory networks (GRNs), observational data alone is not sufficient for accurate graph construction. Saremi et al. [9] proposed an iterative refinement algorithm for extracting gene regulatory networks using random forest, and Omranian et al. [10] applied a joint Lasso algorithm for the single causal graph identification.

While the aforementioned approaches focus on learning a single graph by combining multiple datasets, we discuss a different scenario in our paper: we have multiple datasets and are learning a (potentially unique) graph for each dataset. There are also other works that study the use of heterogeneous data in non-causal settings that inspired our work, including sample-specific disease correlation networks [11] and sample-specific predictive models [12].

### Data-driven algorithms for learning single causal graph

The complexity and dimensionality of biomedical data require methodologies that integrate existing biological knowledge with patient-specific data, such as knowledge graphs [13, 14]. For example, CLinical Embedding of Patients (CLEP) [15] incorporates patient-level multi-omics data into a knowledge graph to model the underlying relationships between patients and clinical features for identifying Alzheimer’s patients and their properties. Medical knowledge graphs or deep architectures that utilize patient-level medical data are important for developing accurate and generalizable clinical decision-support models [15–22]. Moreover, the utilization of a personalized biomedical graph enables the identification of patient-specific biological mechanisms [7, 15, 23, 24], offering further insights into the causal relationships in specific diseases or patient subgroups. Existing data-driven algorithms for learning single causal graphs can generally be categorized into two groups: score-based and constraint-based learning methods.

Score-based methods for learning directed acyclic graphs (DAGs) design specific scores for evaluating DAGs, typically penalized data likelihood, and then find the graph with the highest score. Notably, Greedy Equivalent Search (GES)[25] and its variants work by iteratively adding and deleting single edges to identify the DAG with the best (penalized) data likelihood. The recent trend in DAG learning has been to focus on solving a continuous relaxation of the problem. NOTEARS [7] was the first in this line of work. The paper proposed a novel constraint for the graph represented by an adjacency matrix to be a DAG. Based on this formulation, the paper then designed a constrained optimization problem for identifying a DAG. Follow-up work also utilized the DAG constraints to reduce the search space of possible causal graphs. For example, in DAG-GNN [26], the authors proposed to model DAG in an encoder-decoder framework, where the weighted adjacency matrix is a variable explicitly used in both the encoder and decoder. Variational inference algorithms are then used to maximize the data likelihood for learning the adjacency matrix. On the other hand, GraN-DAG [27] models the relationship of a variable with its parents using neural networks, and the summation for all network weights is defined as the corresponding entry in the weighted adjacency matrix.

Another category of DAG learning methods is constraint-based. A typical assumption of these methods is that there is a one-to-one correspondence between the conditional independence between variables from the observed data distribution and the missing edge in the DAG, i.e., the faithfulness assumption. For example, PC algorithm [28], which is named after its inventor Peter Spirtes and Clark Glymour, and its variants, such as fast causal inference (FCI), use this assumption to first identify an undirected skeleton of the variable relationships, then orient the edges to obtain the DAG. Other work studies how to combine score- and constraint-based DAG learning, as seen in the max-min hill climbing algorithm [29].

### Meta-learning

Meta-learning [30] is a machine learning paradigm for learning from a set of (training) tasks to adapt faster to a new (test) task. Meta-learning has seen great success recently in few-shot learning and reinforcement learning domains. There are two major types of algorithms in meta-learning. Model-based approaches, such as meta-long short-term memory [31], propose to learn a meta-model that outputs the model parameters based on the input dataset. Model-agnostic approaches [32, 33] propose to learn the initialization of model parameters so that the adaptation can be faster with a well-tuned initialization.

## Method

**Fig. 2:**
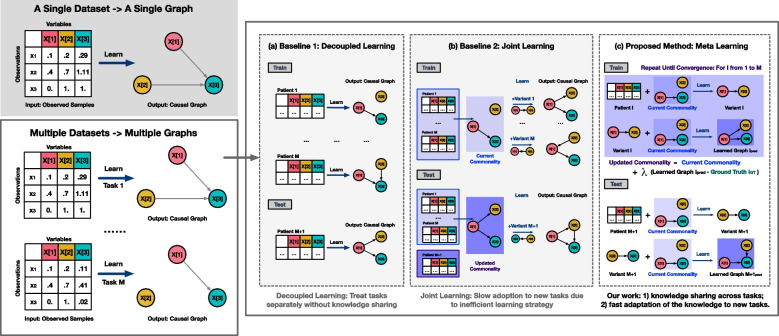
An illustration of different learning schema for multi-task causal graph learning. **a** Decoupled learning methods solve each task independently without considering the similarities among tasks; **b** Joint learning methods introduce an additional set of parameters to model the shared commonality among all tasks and learn a separate variation parameter for each task; and **c** Our method applies a new framework based on meta machine learning principle and learns both commonality and variations for each task. Compared with baselines, meta-learning enables the sharing of knowledge across tasks for enhanced prediction performance and the rapid adoption of knowledge to new tasks for efficient causal graph learning

In this section, we present our framework using meta-learning for learning personalized causal graphs in biomedicine. Firstly, we present the preliminaries of single-task causal graph learning, which serves as the foundation for constructing a biomedical graph from individual patient data. Subsequently, we illustrate our unique multi-task causal graph learning setting, which extracts common patterns from multiple patient graphs and applies this information to develop individualized graphs. Lastly, we introduce our proposed approach for meta-causal structure learning for updating shared common knowledge. In multi-task causal graph learning, the proposed optimized initial guess of shared commonality enables the rapid adoption of knowledge to new tasks for efficient causal graph learning. An overview of the proposed multi-task causal graph development is available in Fig. 2.

### Preliminaries: single-task causal graph learning

In the single task causal structure learning (Fig. 2), we are given an observation data matrix for a patient, which contains *N* samples from a *D*-dimensional space, $\varvec{X} \in \mathbb {R}^{N \times D}$, and our goal is to learn the causal relationships between the *D* variables, $\varvec{X}[1], ..., \varvec{X}[D]$. Here we focus on the binarized causal relationships, i.e., whether a causal relationship exists between $\varvec{X}[d_1]$ and $\varvec{X}[d_2]$, for $d_1 \ne d_2 \in \{1, ..., D \}$. Correspondingly, this is the same as a graph $\mathcal {G}$, where an edge $\varvec{X}[d_1] \rightarrow \varvec{X}[d_2]$ indicates the existence of causal relationship between variable $\varvec{X}[d_1], \varvec{X}[d_2]$. A causal graph $\mathcal {G}$ corresponds to an adjacency matrix $\varvec{W} \in \mathbb {R}^{D \times D}$, and the non-zero entries of $\varvec{W}[d_1, d_2]$ indicate the edge $\varvec{X}[d_1] \rightarrow \varvec{X}[d_2]$.

A single causal structure learning algorithm defines a procedure to output $\varvec{W}$ from $\varvec{X}$, $\mathcal {A}(\varvec{X}, \gamma _0) \rightarrow \varvec{W}$. Here we use $\gamma _0$ to denote our prior or domain knowledge of the underlying causal structures. For example, we can constrain the graph to be directed acyclic graphs (DAGs), or we can learn from prior intervention experiments that certain edges exist or do not exist. In our previous example in Fig. 1, we have an observation in the form of $\varvec{X} \in \mathbb {R}^{N \times 5}$, and the goal is to find the causal relationship between the five variables: $\varvec{X}[1] - \varvec{X}[5]$.

A popular method is to use a score-based formulation for the single task causal graph learning problem $\mathcal {A}(X, \gamma _0) \rightarrow \varvec{W}$

1 $$\begin{aligned} \varvec{W} = \arg \max Score(\varvec{X}, \varvec{W}) + \lambda Reg(\varvec{W}). \end{aligned}$$

The score function is the maximum likelihood of observing $\varvec{X}$ given $\varvec{W}$. Here we assume a linear model of likelihood, $\frac{1}{2N} \left\Vert {X - XW}\right\Vert _F^2$, but other types of assumptions can also be encoded in the loss function. We impose additional constraints using a regularization function. For example, we can use L1 norm to constrain the number of learned edges on $\varvec{W}$ [34], $\left\Vert {W}\right\Vert _1 = \left\Vert {vec(W)}\right\Vert _1$. In some cases, our prior knowledge specifies the graph corresponding to $\varvec{W}$, denoted as $G(\varvec{W})$ is a DAG, so we can also impose additional acyclic constraints, such as those proposed in [7].

2 $$\begin{aligned} J(\varvec{W}) = \frac{1}{2N} \left\Vert {\varvec{X} - \varvec{X} \varvec{W}}\right\Vert _F^2 +{} & {} \lambda \left\Vert {\varvec{W}}\right\Vert _1 \quad \nonumber \\{} & {} s.t. G(\varvec{W}) \in DAGs \end{aligned}$$

### Problem formulation: multi-task causal graph learning

In multi-task settings, instead of a single data matrix from a single patient, we now have matrices from multiple patients. Each patient has their own causal graph $\varvec{W}^{(m)}$ and a corresponding observational data matrix $\varvec{X}^{(m)}$. Each patient network becomes a single-task causal structure learning problem $\mathcal {A}\left(\varvec{X}^{(m)}, \gamma _0\right) \rightarrow \hat{\varvec{W}}^{(m)}$.

For this collection $\mathcal {D} = \left\{ \mathcal {A}\left(\varvec{X}^{(m)}, \gamma _0\right) \rightarrow \hat{\varvec{W}}^{(m)} \right\}_{m=1, ...}$, our goal in the multi-task setting is two-fold: 1) to identify the causal structure correctly for each $\varvec{W}^{(m)}$’s and 2) to extract useful knowledge $\gamma _0$ that reflects the shared common knowledge for future causal structure learning tasks.

**Fig. 3:**
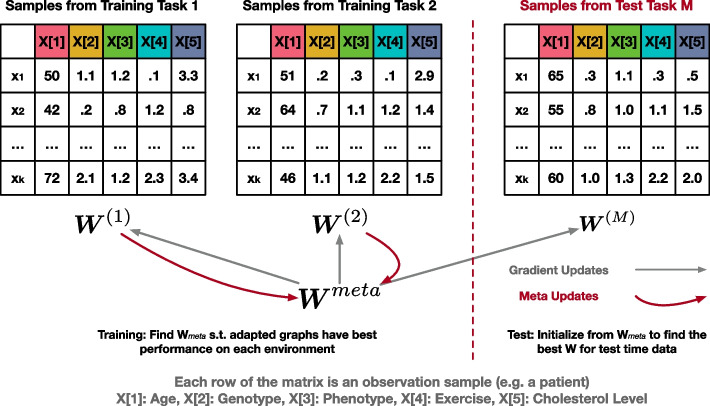
An illustration of proposed causal graph learning framework using meta machine learning. We amortize the knowledge in the initialization graph for the causal structure learning problem $\varvec{W}^{meta}$. For each task, we solve the structure learning problem with initialization from $\varvec{W}^{meta}$. We also update this knowledge after solving each training task via meta-learning principles. For test task unseen, we adapt our knowledge to this task but do not update the knowledge

We will use superscript ^(m)^ to denote the *m*th task, while lowerscript _i_ denotes the *i*th sample. Similar to the conventional supervised learning setting, we have a ‘train’ and ‘test’ phase. As illustrated in Fig. 3, during training, we are given a collection of patients/tasks $\mathcal {D}_{train} = \left\{ \mathcal {A}^{(m)} \right\}_{m=1, ..., M_{train}}$, and for each patient $\mathcal {A}^{(m)}$ we learn the causal graph. In parallel, we also update $\gamma _0$ as we solve each of the tasks so when we encounter a new task we can quickly solve it based on our aggregated knowledge $\gamma _0$ to improve the performance, i.e., we optimize the average performance on the $M_{test}$ test set.

3 $$\begin{aligned} \frac{1}{M_{test}}\sum \limits _{m=1}^{M_{test}} Performance\left( \hat{\varvec{W}}^{(m)} , \varvec{W}^{(m)}\right) \nonumber \\ \text {with}~~ \hat{\varvec{W}}^{(m)} = \mathcal {A}\left( \varvec{X}^{(m)}, \gamma _0\right) \end{aligned}$$

The performance of our algorithm is evaluated on an unseen test set $\mathcal {D}_{test} = \left\{ \mathcal {A}^{(m)} \right\}_{m=1, ..., M_{test}}$, when we make use of our learned $\gamma _0$.

### Proposed algorithm: meta causal structure learning

We then utilize meta-learning principles [32] to formalize our intuition of updating our shared knowledge $\gamma _0$. Specifically, we adopt an explicit approach to address the problem by focusing on fine-tuning a model using a gradient-based optimization method for new tasks. Our objective is to train a model that can quickly adapt to new tasks from a specific task distribution. We achieve this by identifying model parameters that are highly sensitive to changes in the task. The overview of meta training algorithm is available in Algorithm 1. Importantly, our methodology is not dependent on any particular model structure (i.e., model-agnostic). Instead, it is based on the key assumption of model-agnostic meta-learning that a good initialization of the parameters helps the optimization algorithm reach the final solution faster. By updating this initialization, we can share the knowledge across different tasks and facilitate learning of similar tasks.

**Figure:**
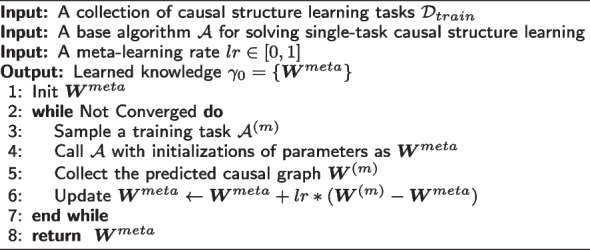
**Algorithm 1** Algorithm for Meta Training

To learn a good initialization of the parameters for model optimization, we utilized a first-order update rule (as shown in Line 6 in Algorithm 1), Reptile [35], as the meta update rule. When facing a new task at test time, it optimizes model parameters by generalizing from only a small number of examples. Specifically, instead of simply updating $\varvec{W}^{meta}$ in the direction $\varvec{W}^{(m)} - \varvec{W}^{meta}$, we can treat $\left(\varvec{W}^{(m)} - \varvec{W}^{meta}\right)$ as a gradient and plug it into an adaptive algorithm. When $lr=0.$, we always start from the same initialization graph, and there is no knowledge sharing among different tasks. While when $lr=1.$, we are continuously learning from the previous ending stage, similar to the fine-tuning practice in the computer vision domain. By selecting a learning rate between [0, 1], we find a trade-off between the current task and all the seen tasks. During test time, we iterate through all our test tasks using Line 5 without updating our $\varvec{W}^{meta}$.

## Results

In this section, we present how we applied our proposed causal learning optimization algorithms to improve the performance of multi-task causal graph learning models on real-world and synthetic datasets. For real-world data, we choose a common benchmark causal graph learning dataset with gene expression levels to understand the applicability of our algorithm to real-world biomedical data. To understand our algorithm’s performance under different scenarios and the impact of factors such as task difficulty and sample efficiency, we also experimented with synthetic datasets to control the data generation process. We varied the number of tasks, the data distribution of each task, and the sample size of each task.

### Dataset

#### Synthetic dataset

For the purpose of data simulation, we generate causal graphs and their corresponding observational data. We utilize two types of random graph models: Erods-Renyi (ER) graphs and scale-free (SF) graphs. Based on the graph, we generate *N* observation samples. In our experiments, we examine two sample cases: a small sample case with $N=50$ and a large sample case with $N=500$. Table 1 summarizes the four configurations. Subsequently, we apply multi-task causal structure learning algorithms to the collection of *M* tasks and report the average accuracy on the *M* graphs. Using our running example, we set the number of patients as $M=50$. We also vary the number of nodes *d*. For each patient, we generate a random causal graph with a specified number of nodes in the graph *d*, where the expected number of edges is 3*d*. This study serves extensive examinations for two purposes: firstly, to evaluate the performance of algorithms under different conditions, and secondly, to analyze how the performance of each algorithm changes as the number of variables (*d*) and the task difficulty increase.

**Table 1:** Configurations of the synthetic datasets generated using ER and SF graph models

Configuration	Graph type	Sample size (N)
1	Erdös-Rényi (ER)	50
2	Erdös-Rényi (ER)	500
3	Scale-Free (SF)	50
4	Scale-Free (SF)	500

#### Real-world dataset

We adopt the SACHS dataset [36], a common benchmark in the causal inference literature, as a real-world application for further evaluation of the proposed causal inference framework. The Sachs dataset measures the level of protein and phospholipid expression in human cells. It contains 7466 continuous measurements of protein and phospholipids expression levels in 11 human immune systems cells, i.e., we are learning a causal graph of 11 nodes. In addition to the original cell type, there are also 13 different interventions (e.g., inhibition of PKC isozymes). A detailed description of the specific interventions is available in [36]. Thus, we have a total of 14 related tasks. We use 9 tasks for training and 2 for testing, and the results are averaged over 10 folds.

### Training and inference

In each scenario, we have a set of variables with unknown causal relationships. We also have a collection of training tasks and another collection of test tasks, with each task being a causal graph learned from observational data. During training, each algorithm receives all training tasks. During testing, we evaluate the performance of each algorithm by computing the average performance metrics of the algorithm over all test tasks.

### Evaluation metrics

For causal models described with adjacency matrices, we evaluate how close the fitted $\hat{W}$ is to the true model *W* (assuming we have access to ground-truth data), or how well $\hat{W}$ describes the observed data. We compute the metrics for each of the test tasks and report their average as the final evaluation for the multi-task setting of causal graph learning.

#### Classification-based metrics

For the true model *W* with *d* variables, there are $\frac{d(d-1)}{2}$ edges. We can regard each of the edges as a binary classification problem. Thus, for a collection of $\frac{d(d-1)}{2}$ problems, we can define true positive rate (TPR), false discovery rate (FDR), and false positive rate (FPR). As we are dealing with DAGs, we can calculate the following:

- True positive (TP): A predicted edge is in the correct direction
- Reverse (R): for a true edge, the predicted direction is reversed
- False positive (FP): the predicted edge doesn’t exist in the undirected skeleton of the true graph

#### Structural hamming distance

Structural Hamming Distance (SHD) is defined based on the popular Hamming distance. Briefly, for two graphs $W^{(1)}, W^{(2)}$, SHD is the number of graph edits (edge insertion, deletion, or flips) required to make the two graphs identical [7].

#### Number of non-zero entries

In addition, we also report the number of non-zero entries (NNZ) in our prediction. SHD alone is not sufficient for evaluation. For example, in a graph of 10 edges, a predicted graph with 0 edges and 20 edges (10 true edges plus 10 edges) can both have an SHD of 10. In this case, we would prefer the graph with more edges in exploratory studies where we want to validate the causal relations from our algorithmic analysis.

### Baselines

We will compare meta-learning-based causal graph learning algorithms against conventional causal graph learning and other multi-task algorithms.

#### Decoupled learning algorithms

Specifically, we choose two decoupled learning algorithms. In decoupled learning, the algorithm solves each task separately and does not extract shared network structures. During testing, each test task is treated separately. We use the state-of-the-art causal graph learning algorithm NoTears-L1 [7]. In addition, we use an L1 regularized causal graph learning algorithm. As this algorithm does not impose the directed acyclic graph constraints as NoTears-L1 does, we call this algorithm Unconstrained-L1.

#### Multi-task learning algorithms

We also study a conventional multi-task learning algorithm combined with NoTears-L1. For each test task *i*, we learn its corresponding causal graph as $W^i = W0 + \Delta ^i$, where *W*0 is the baseline causal model shared across all tasks, and $\Delta ^i$ is the task-specific parameter. For a total of $M_{test}$ tasks, we are now solving an optimization problem the size of $M_{test} + 1$. In practice, this algorithm fails to scale with increasing test size. For our meta-learning-based framework, we plug in both the decoupled NoTears-L1 and Unconstrained-L1 algorithms and name them MetaNoTears-L1 and MetaUnconstrained-L1, respectively.

### Main results on synthetic data

**Fig. 4:**
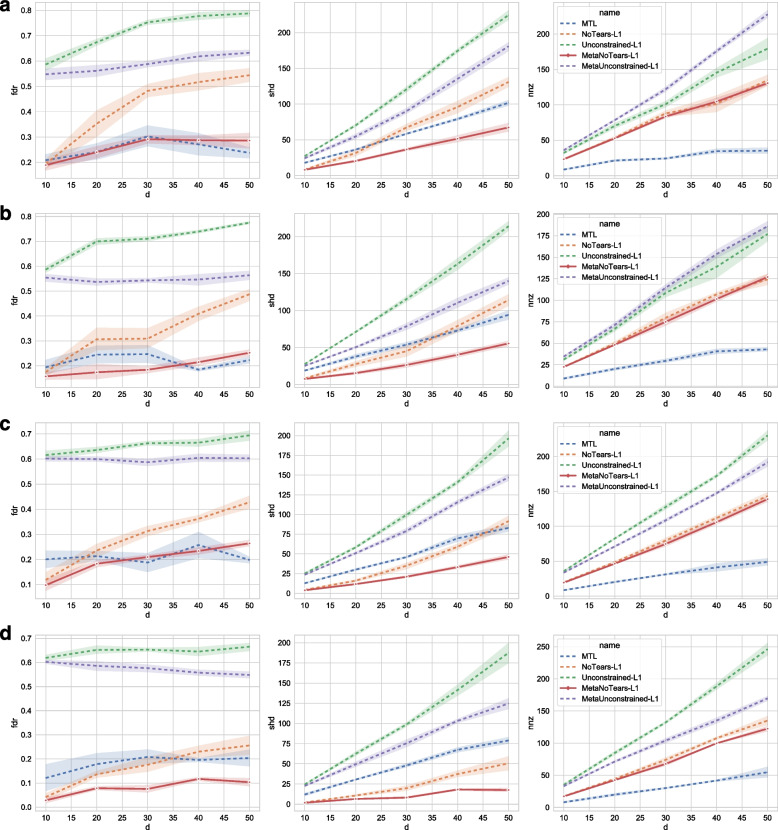
Overall results: The X axis denotes the size of the graph *d*, and the Y axes are the three types of evaluation metrics, FDR (false discovery rate), SHD (structural Hamming distance), and NNZ (number of non-zero entries). When we fix the number of samples per patient/task and the graph type, we can see that MetaNoTears-L1 outperforms others in SHD consistently. Although MTL has a similar performance with MetaNoTears-L1 on FDR, it has a higher SHD and also predicts a much lower number of edges. In addition, as the size of the graph increases, MetaNoTears-L1’s performance worsens slowest

In this section, we present the performance comparison of our algorithms against baselines, as shown in Fig. 4. This graph shows how the performance of all five algorithms (MultiTaskLearning NoTears-L1, Single-Task NoTears-L1, Single-Task Unconstrained-L1, MetaLearning-based MultiTask NoTears-L1, and MetaLearning-based MultiTask Unconstrained-L1) changes with respect to the changing number of nodes in the graph (d in the X-axis). The three subfigures from left to right show the performance metrics: FDR, SHD, and the number of NNZ.

For FDR, we can see that our MetaNoTears-L1 outperforms NoTearsL1, Unconstrained-L1, and MetaUnconstrained-L1 by a large margin; MTL and MetaNoTears-L1 have similar FDR performance in FDR. For SHD, MetaNoTears outperforms all other four algorithms by a large margin consistently, reducing SHD by 50%-75%.

Non-zero entries reflect a trade-off between false positive and false negative rates. It is clear that MTL makes very conservative predictions. For example, the number of predicted edges is the smallest, and it has a rather low FDR. However, this comes at the cost of increasing SHD. When the size of the graph grows, we can see that the SHD of the MTL learned graph also grows, indicating that MTL makes many more mistakes than MetaNoTears-L1.

Overall, we can see that 1) SHD increases as the size of graphs grows, but 2) our algorithm decreases the slowest, and 3) our algorithm maintains a relatively flat FDR.

### Main results on real-world data

In this section, we present the results of our algorithms on real-world data. As we can observe from Table 2, when we compare a base algorithm with its meta-learning counterparts we proposed (i.e., Unconstrained vs. MetaUnConstrained-L1, NoTears-L1 vs. MetaNoTears-L1) and the state-of-the-art methods like AVICI [37], meta-learning versions improve over their counterparts and existing methods. In addition, the NoTears-L1 algorithm performs better than the UnConstrained algorithm in the Sachs dataset. Overall, MetaNoTears-L1 is the best-performing algorithm and shows improvement in the four metrics. As the graph only has 11 nodes, we are not observing a significant improvement in the SHD distance, but still, we can see that our algorithm improves significantly over NoTears on the false discovery rate and false positive rate. We perform additional results on synthetic data to further understand the performance of our algorithms against others with larger-scale data.

**Table 2:** Main results on the real-world dataset, SACHS. Unconstrained versions of causal discovery learning yield relatively low performance, while the NoTears algorithms are the two best. MetaNoTears-L1 is the best-performing algorithm and shows improvement in the metrics. Abbreviations: FDR (false discovery rate), TPR (true positive rate), FPR (false positive rate), SHD (structural Hamming distance), and NNZ (number of non-zero entries)

Methods	FDR	TPR	FPR	SHD	NNZ
MTL	0.73±0.01	**0.42±0.03**	0.44±0.02	20.24±0.73	23.92±0.95
NoTears-L1	0.69±0.04	0.34±0.06	0.31±0.01	16.83±1.76	17.50±0.32
Unconstrained-L1	0.88±0.02	0.14±0.04	0.40±0.02	26.36±0.52	17.96±0.99
AVICI	0.80±0.02	0.40±0.05	0.35±0.03	20.23±1.32	18.23±0.47
MetaUnconstrained-L1	0.98±0.01	0.02±0.01	**0.28±0.00**	26.20±0.73	11.32±0.18
MetaNoTears-L1	**0.62±0.02** ^∗^	0.40±0.04 *n*.*s*.	**0.28±0.01** ^∗^	**15.50±0.50** *n*.*s*.	17.17±0.29

*denotes the significance of 0.05

### Sample efficiency

**Fig. 5:**
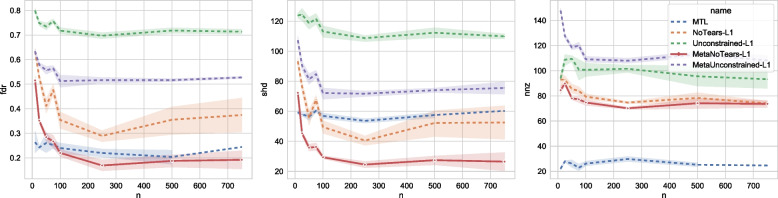
Impact of sample size on each of the algorithms on a synthetic dataset. The X-axis denotes the size of the graph *d*, and the Y axes are the three types of evaluation metrics, FDR (false discovery rate), SHD (structural Hamming distance), and NNZ (number of non-zero entries). We fix the number of patients to 50, the size of the graph to 30, and the type of graph to be Erdos-Reni. We vary the number of samples per patient from 10 to 750 and plot how each algorithm’s performance changes accordingly. For FDR, we can see that MTL and MetaNoTears-L1 outperform the other three algorithms by a large margin. Between MTL and MetaNoTears, when the number of samples is smaller than 100, MTL has a better performance. This is because MTL makes much more conservative actions, as shown by the NNZ graph on the right. After the number of samples increases over 100, MetaNoTears-L1 starts to outperform MTL by 0.05. For SHD, we can see that except for the case when the number of samples is 10, MetaNoTears-L1 consistently outperforms other algorithms significantly, improving the SHD by 50% - 90%

In this section, we present the impact of sample size on each of the algorithms. We use an ER graph and fix the number of variables (*d*) to be 30 and select 90 edges. We also set the total number of patients to 50. We then vary the number of samples per patient (*N* from 10 to 750. We observe in Fig. 5 that FDR we see that MTL and MetaNoTears-L1 outperform the other three algorithms by a large margin. Between MTL and MetaNoTears, when the number of samples is smaller than 100, MTL has a better performance. This is because MTL makes many more conservative actions relative to MetaNoTears, as shown by the NNZ graph on the right. After the number of samples increases over 100, MetaNoTears-L1 starts to outperform MTL by 0.05. For SHD, we see that except for the case when the number of samples is 10, MetaNoTears-L1 consistently outperforms other algorithms significantly (e.g., improving the SHD by 50% - 90%).

### Number of task

**Fig. 6:**
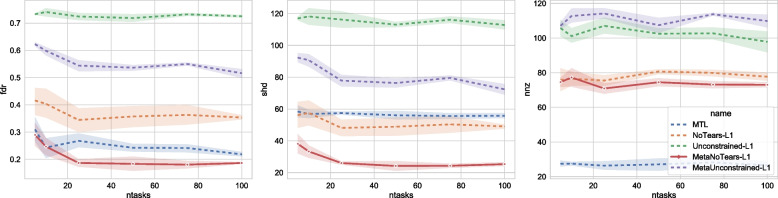
Impact of the number of tasks in training set on each of the algorithms on a synthetic dataset. The X-axis denotes the size of the graph *d*, and the Y axes are the three types of evaluation metrics, FDR (false discovery rate), SHD (structural Hamming distance), and NNZ (number of non-zero entries). We fix the size of the graph to be 30, and the type of graph to be Erdos-Reni, the number of samples to be 100. We vary the number of patients from 10 to 100 and plot the performance against it. When the number of tasks is smaller than 30, adding more tasks to the problem increases the algorithms’ performance. This is because increasing more similar tasks also increases the number of samples for each algorithm. However, after the number of tasks is greater than 40, adding more tasks does not have an additional benefit for MetaNoTears-L1. Among the five algorithms, our algorithm has the lowest SHD and FDR (except for FDR when the number of tasks = 10), while predicting considerably more edges and closer to the true graph compared to MTL

We can observe the impact of the number of tasks in the training set on the algorithms’ performance from Fig. 6. When the number of tasks is smaller than 30, adding more tasks to the problem increases the algorithms’ performance. This is because increasing more similar tasks also increases the number of samples for each algorithm. However, after the number of tasks is greater than 40, adding more tasks does not have an additional benefit for MetaNoTears. Among the five algorithms, our algorithm has the lowest SHD and FDR (except for FDR when the number of tasks = 10), while predicting considerably more edges and being closer to the true graph compared to MTL.

### Sensitivity to hyper-parameters

In this section, we explore the impact of hyperparameters on the performance of our algorithms. The hyperparameters we consider are the meta-learning rate (lr) and the number of outer loop steps. We use the MetaNoTears version of our algorithm, and test on a 30-node ER graph with 500 samples, and test different scenarios.

#### Meta learning rate

**Fig. 7:**
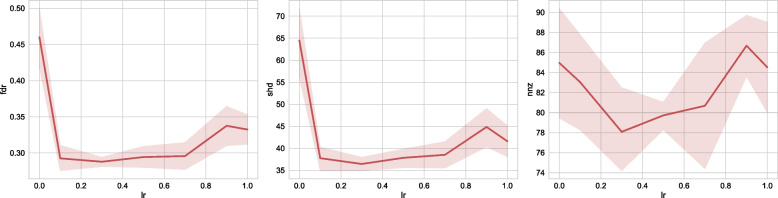
Sensitivity to meta-learning rates. We choose NoTears-L1 as the base algorithm, and study our algorithm’s sensitivity to meta-learning rates (X-axis). We test on a 30-node ER graph with 500 samples. Here the case where meta-learning rate = 0. corresponds to the NoTears-L1 algorithm without the meta-learning framework. When meta-learning rate = 1., for a new patient, the algorithm continuously starts from the previous patient’s result. When meta-learning rate is in the range of [0.1, 0.7], the MetaNoTears-L1 algorithm finds a good balance between learning knowledge from past patients and adaptation to new patients

Here $lr=0.0$ corresponds to the decoupled learning algorithm NoTears, while $lr=1.0$ corresponds to continuous learning. As we can observe in Fig. 7, in terms of FDR and SHD, using meta-learning algorithm (setting $lr > 0$) increases the performance of the algorithm compared to the decoupled NoTears. This again verifies the benefit of knowledge sharing enabled by our meta-learning algorithm. As the learning rate increases to sufficiently large (greater than 0.8 in this case), we can see that the algorithm performance decreases. This is because the algorithm overfits one particular dataset and “forgets” the previously encountered tasks.

#### Number of outer loop steps

**Fig. 8:**
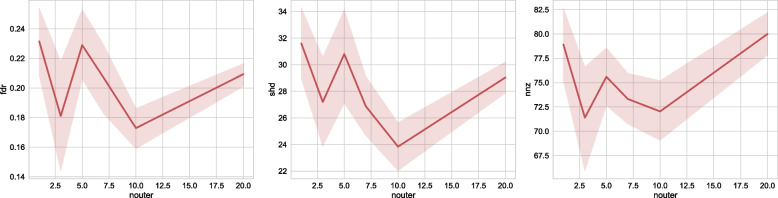
Sensitivity to the number of outer steps. We choose NoTears-L1 as the base algorithm, and study our algorithm’s sensitivity to the number of outer steps (X-axis). We test on a 30-node ER graph with 500 samples: the algorithm’s performance is much noisier with respect to changing numbers of outer steps. Thus, in practice, we need to carefully select the number of outer steps for each different dataset

We can observe from Fig. 8, that the number of outer steps influences the final performance slightly. We can observe that in this experiment, 10 outer steps yield the best performance in FDR and SHD. Increasing the number of outer steps further, however, won’t increase the performance correspondingly. Thus, in practice, we should select a suitable number for the outer steps, for example, through grid search over the possible range.

#### $L_1$ regularization

**Fig. 9:**
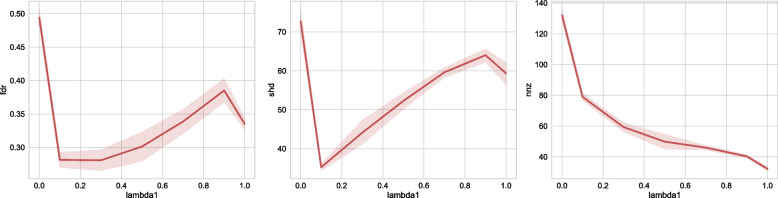
Sensitivity to L1 regularization. The increasing strength of L1 regularization reduces the number of non-zero entries identified, and choosing the number to be 0.1 in practice gives us a good trade-off between the false discovery rate and the number of non-zero entries

We have also experimented with varying the L1 regularization strength from 0.0 to 1.0 on a linear scale, as shown in Fig. 9. The result is in line with the L1 regularization effect on other models such as Lasso regression. Increasing the regularization strength will lead to fewer identified edges. In practice, we also use 0.1 as the regularization hyperparameter value.

## Discussion

As biomedical causal graphs are often heterogeneous, the multi-task learning approach for causal graph problems is very common and challenging due to the low sample sizes. While the existing approaches focus on learning a single graph by combining multiple datasets (Table 3), we discuss a different scenario in our paper: we have multiple datasets and are learning a (potentially unique) graph for each dataset. In this paper, we adapted a meta-machine learning algorithm that can process the data from all tasks to identify shared knowledge. The shared knowledge, in turn, can lead to new discoveries of personalized graphs faster and more accurately.

**Table 3:** Comparison of existing graph representation and causal discovery works in biomedicine with heterogeneous patient data

Reference	Task	Graph type	Single-task	Multi-task
Rocheteau et al. [19]	outcome prediction	undirected graph	yes	no
Lu et al. [20]	disease prediction	bipartite or undirected graph	yes	no
Li et al. [21]	CTR prediction	weighted fully-connected graph	yes	no
Xu et al. [22]	clinical prediction	hypergraph	yes	no
Zheng et al. [7]	biomedical structure	directed acyclic graph	yes	no
**Ours**	**causal discovery**	**directed acyclic graph**	**yes**	**yes**

**Fig. 10:**
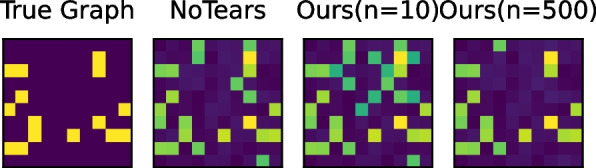
Visualization of Learned Graphs. We visualize the graphs learned from different algorithms. As shown in the figure, with an increasing number of tasks (from 5 to 10), our algorithm can utilize additional data to increase its performance, resulting in fewer false positive edges. In the depicted adjacency matrices, a dark hue signifies a value of 0, while a bright yellow indicates a value of 1. The various shades of green represent the probability estimates produced by each algorithm

As we show in the synthetic and real-world datasets, in all five scenarios with different underlying data distributions and dataset sizes, our algorithm (meta-learning-based causal structure learning) consistently outperforms state-of-the-art approaches. This shows the general applicability of our algorithm to solve the challenging multi-task causal structure learning problem. The key ingredient for our algorithm is to extract common knowledge from different tasks so that we can adapt to unseen tasks at test time. Case studies in Figs. 5 and 6 studying the impact of sample size on multi-task causal structure learning problems to further understand the behavior of our algorithm against others. Moreover, we also discuss the sensitivity of our algorithms to hyper-parameters to demonstrate the robustness of our proposed causal graph learning strategy. In addition, we also analyze the graphs generated by various algorithms in Fig. 10. When the number of tasks increases (from 5 to 10), our algorithm effectively utilizes more data, improving its performance and reducing the number of false positive edges.

Our algorithm improves multi-task causal graph learning in multiple ways compared to state-of-the-art multi-task causal graph learning algorithms. Decoupled learning algorithms such as [7] learn each personalized graph separately. Although they are faster, they fail to capture the commonality shared among all tasks. Joint learning algorithms such as conventional multi-task learning algorithms [38] solve all personalized causal graph learning simultaneously. These algorithms are less efficient: 1) they have higher time and space complexity, for M patients, the time and space of complexity is O(M) to that of the decoupled learning and our algorithm; 2) if we want to learn a new task, these algorithms need to learn from all existing tasks plus this new task simultaneously again, i.e., solving a problem the size of M+1. On the other hand, our algorithm only needs to start from our initial guess and solves a single task, which is much more time and space efficient.

Experiments on both real-world and synthetic graphs show that our framework can improve upon baseline algorithms, such as the state-of-the-art decoupled NoTears algorithm and joint multi-task learning algorithms. Specifically, in graph learning performance (measured by FDR and SHD), our algorithm MetaNoTears-L1 outperforms others, with a 50%-75% reduction in SHD and 0.2 - 0.3 reduction in FDR while identifying 50% more edges than the second-best performing algorithms. Our algorithm works as a meta-algorithm: it can incorporate different single-task causal graph learning to extract common knowledge shared among patients. For two of the single-task causal graph learning algorithms, comparing Unconstrained to MetaUnConstrained-L1 and NoTears-L1 to MetaNoTears-L1, our algorithm improves upon its single-task counterpart by 50% percent in FDR and SHD. This shows the benefit of common knowledge extraction achieved by our framework. When we fix the number of samples and increase the number of nodes on the graph, the problem becomes more challenging. Compared to baseline algorithms, our MetaNoTears-L1 has the lowest error overall, and the error increases at the slowest rate. When we fix the number of nodes on the graph and increase the number of samples per graph, our algorithm MetaNoTears-L1 has the lowest error overall. When the number of samples per graph is small, the error of our algorithm decreases at the fastest rate. This shows that our algorithm can make better use of the additional samples per graph. When we change the number of tasks, all algorithms have a relatively flat change in performance when the number of tasks is greater than 20. Our algorithm also has the lowest error overall.

Specifically, for each experiment among the three dimensions (i.e., number of variables in the causal graphs, number of patients/tasks, and number of observational data points for each patient), we fix two dimensions vary the other, and then observe how causal graph learning metrics on test tasks change, our algorithm has shown major improvement in graph accuracy in comparing to state-of-art algorithms: (1) When we fix the number of tasks and the number of samples per task and vary the number of variables in the graph. Our algorithm consistently reduced the false discovery rate by 10-20% compared to the state-of-the-art single-task causal graph learning algorithm and the graph edit distance by 50-70%; (2) When we fix the number of variables in the graph and the number of tasks and vary the number of samples per task, our algorithm reduced the false discovery rate by 5-10% and the graph edit distance by 40-75%; and (3) When we fix the number of variables in the graph and the number of samples per task, and vary the number of tasks, our algorithm reduced the false discovery rate by 5-10% and the graph edit distance by 33-55%.

Building personalized causal graphs for each individual poses a significant challenge due to the limited data available per patient. In this study, we introduce a novel algorithmic framework that leverages meta-learning for the multi-task learning of personalized causal graphs in biomedicine. Unlike previous studies that concentrated on learning causal graphs from single patients, our approach efficiently extracts common patterns across multiple patient graphs to construct individualized graphs. It also demonstrates the effectiveness of meta-learning in personalized causal graph learning and cause inference modeling for biomedicine. One potential limitation is the relatively limited sample size, which may impact the robustness and generalizability of the learned causal graphs. Additionally, handling complex biomedical data efficiently can pose challenges in scenarios where computational resources are constrained or when processing exceedingly large datasets. In our future work, we will optimize the applicability of our proposed methods across diverse biomedical data. This will allow for more efficient and effective integrated analyses across various data distributions, including datasets from different clinical institutions.

## Conclusion

This paper is a first step towards solving multi-task learning problems more efficiently in learning personalized causal graphs. Our study shows the possibility to extract knowledge from different tasks to facilitate the learning of new unseen tasks, and we believe this opens up the possibility for future lines of research. The algorithm we proposed can help analyze heterogeneous datasets with multi-task structures. It also has the potential to be used to establish personalized causal graphs of cancer patients’ gene expression levels or of brain connectivity via imaging data. We can also apply this principle to other biomedical data analytic settings. For example, we may be able to infer the personalized treatment effects of drugs on patients. For future work, we aim to further study how we can improve the current multi-task causal graph learning problems via advanced meta-learning approaches, for example, model agnostic meta-learning and Bayesian meta-learning algorithms. We can also apply our algorithms to other settings, for example, integrating datasets from different clinical institutions and from different countries, and making customized clinical decisions and personalized treatment.

## Data Availability

No datasets were generated or analysed during the current study.
